# Immunogenicity of bacterial-expressed recombinant *Plasmodium knowlesi* merozoite surface protein-1_42_ (MSP-1_42_)

**DOI:** 10.1186/1475-2875-12-454

**Published:** 2013-12-19

**Authors:** Fei Wen Cheong, Mun Yik Fong, Yee Ling Lau, Rohela Mahmud

**Affiliations:** 1 Department of Parasitology, Faculty of Medicine, University of Malaya, 50603, Kuala Lumpur, Malaysia; 2 Tropical Disease Research and Education Centre (TIDREC), Faculty of Medicine, University of Malaya, 50603, Kuala Lumpur, Malaysia

**Keywords:** *Plasmodium knowlesi*, Merozoite surface protein, Recombinant expression, Immunogenicity

## Abstract

**Background:**

*Plasmodium knowlesi* is the fifth *Plasmodium* species that can infect humans. The *Plasmodium* merozoite surface protein-1_42_ (MSP-1_42_) is a potential candidate for malaria vaccine. However, limited studies have focused on *P. knowlesi* MSP-1_42_.

**Methods:**

A ~42 kDa recombinant *P. knowlesi* MSP-1_42_ (pkMSP-1_42_) was expressed using an *Escherichia coli* system. The purified pkMSP-1_42_ was evaluated with malaria and non-malaria human patient sera (n = 189) using Western blots and ELISA. The immunogenicity of pkMSP-1_42_ was evaluated in mouse model.

**Results:**

The purified pkMSP-1_42_ had a sensitivity of 91.0% for detection of human malaria in both assays. Specificity was 97.5 and 92.6% in Western blots and ELISA, respectively. Levels of cytokine interferon-gamma, interleukin-2, interleukin-4, and interleukin-10 significantly increased in pkMSP-1_42_-immunized mice as compared to the negative control mice. pkMSP-1_42_-raised antibody had high endpoint titres, and the IgG isotype distribution was IgG1 > IgG2b > IgG3 > IgG2a.

**Conclusions:**

pkMSP-1_42_ was highly immunogenic and able to detect human malaria. Hence, pkMSP-1_42_ would be a useful candidate for malaria vaccine development and seroprevalence studies.

## Background

Malaria is one of the important infectious diseases that causes high global mortality and morbidity. *Plasmodium knowlesi* has recently been recognized as the fifth *Plasmodium* species that can cause malaria in humans [1,2]. *Plasmodium knowlesi* replicates every 24 hours, which is the most rapid replication rate among all human *Plasmodium* species. Quoditian fever, hyperparasitaemia, life-threatening complications and death may occur if the patient remains untreated [3].

Proteins expressed on the surface of *Plasmodium* merozoites are promising targets for malaria vaccine development. Merozoite surface protein 1 (MSP-1) is a high molecular mass protein which undergoes two proteolytic steps to produce several fragments. Primary processing occurs during maturation of merozoites, and the secondary processing occurs during the invasion of merozoites into erythrocytes [4-6]. Proteolytic processing of MSP-1 has been intensively studied in *Plasmodium falciparum*. During the first processing, the *P. falciparum* MSP-1 precursor polypeptide is cleaved into four major fragments of ~83 kDa (MSP-1_83_), 30 kDa (MSP-1_30_), 38 kDa (MSP-1_38_), and 42 kDa (MSP-1_42_) in size. The secondary processing further cleaves the MSP-1_42_ into two fragments, MSP-1_33_ and MSP-1_19_. The soluble MSP-1_33_ sheds from the merozoite surface [7-9], whereas the membrane-bound MSP-1_19_ remains associated with merozoites and is carried into the new erythrocyte during invasion [10,11].

MSP-1_42_ is one of the leading candidates for blood-stage malaria vaccines as it is able to induce protective immune responses [12-14]. Antibodies directed against MSP-1_42_ and MSP-1_19_ can interrupt merozoite invasion *in vitro*[15-17]. Children with naturally acquired immune response to *Plasmodium* MSP-1_19_ are significantly associated with resistance towards malarial infection and clinical manifestations [18], while pregnant women with anti-MSP-1_19_ antibodies are protected against placental infection and infection in infants [19]. Immunization studies using MSP-1_42_ and MSP-1_19_ in animal models such as rodents, mice and primates [20-24] found that protective immune response is elicited during challenge with life *Plasmodium* parasites.

MSP-1_19_-mediated protective responses are mainly responsible for humoral immunity. Low prevalence of T cell responses to MSP-1_19_ is due to limited T cell epitopes on this fragment_._ Protective T cell responses, on the other hand, are induced by epitopes on MSP-1_33_[25-27]. MSP-1_33_ regulates cell mediated responses inducing effector T cells which help in protective B cells response, cytokines production and antiparasitic activity regulation against *Plasmodium* in an antibody-independent manner [28,29]. It is thus more appropriate to include both MSP-1_19_ and MSP-1_33_ fragments in the malaria vaccine design in order to elicit both humoral and cell mediated responses. Therefore, MSP-1_42_ which has both immunodominant B and T cell epitopes, is considered an important and potential vaccine candidate [30,31].

To date, most of the efforts for development of malaria vaccines and human trials are still focus on *P. falciparum*. Phase I human vaccine studies by using *P. falciparum* MSP-1_42_ in USA [32,33], western Kenya [34] and Mali [35] showed high safety, tolerability and immunogenicity, which protective cytokines and antibody responses were detected in the volunteers. However, the raised anti-MSP-1_42_ antibodies were insufficient to inhibit parasite growth up to protection level [36,37] and in a Phase II human trial with Kenyan children, the overall vaccine efficacy was considerably low [38]. Nonetheless, the low level protection elicited by this single antigen vaccine could be enhanced and overcome by multi-antigens vaccine development or addition of other immunostimulants.

Considerable amount of studies on MSP-1_42_ have been carried out on several *Plasmodium* sp. but not much is known about *P. knowlesi* MSP-1_42_, particularly about its immunogenicity. In the present study, a recombinant MSP-1_42_ of *P. knowlesi* (pkMSP-1_42_) was produced and evaluated using ELISA and Western blot assays. Immunogenicity was assessed using the mouse model. Cytokine levels in pkMSP-1_42_-immunized mice were determined and antibody responses were characterized.

## Methods

### Ethics statement

Animal ethic and experiment procedures were approved by University of Malaya Institutional Animal Care And Use Committee (PAR/28/09/2011/CFW). Human ethic was approved by University of Malaya Medical Centre Medical Ethics Committee (MEC Ref. No: 817.18).

### Construction of recombinant plasmid pkMSP-1_42_

*Plasmodium knowlesi* genomic DNA was extracted from a *P. knowlesi*-infected patient blood sample using blood extraction kit (QIAGEN, Hilden, Germany). The *MSP-1*_*42*_ gene was amplified by polymerase chain reaction (PCR) using primer pair MSP1_42__F: 5′ - CGC*GGATCC*GAGAATCACGTGGCTGCATTCA −3′ and MSP1_42__R: 5′ - CGC*GGATCC*CTAGCTGGAGGAGCTACAGAA −3′ based on the sequence of *P. knowlesi* H strain (GenBank accession number XM_002258546). The amplification conditions were as follows: initial denaturing step at 95°C for 4 minutes; 35 cycles at 95°C for 45 seconds, 55°C for 45 seconds, and 72°C for 1 minute; final elongation step at 72°C for 10 minutes. The PCR product was purified and cloned into pCR 2.1-TOPO plasmid vector (Invitrogen, USA), followed by verification using sequence analysis. Restriction enzyme *Bam*HI was used to digest the plasmid at 37°C for three hours, and the digested fragment was ligated with expression vector pRSET A (Invitrogen, USA) at 4°C overnight. The recombinant plasmid was transformed into expression host *Escherichia coli* strain BL21 (DE3) pLysS.

### Expression of pkMSP-1_42_

BL21 (DE3) pLysS cells containing recombinant plasmid pkMSP-1_42_ was propagated in Luria-Bertani (LB) broth containing ampicillin (100 μg/ml) and chloramphenicol (35 μg/ml) at 37°C with shaking until the optical density at 600 nm (OD_600_) reached 0.4-0.6. The culture was induced with 1 mM isopropyl β-D-1-thiogalactopyranoside (IPTG) and allowed to grow for another two hours. The cells were harvested by centrifugation at 5,000 × g for 10 minutes.

### Purification of pkMSP-1_42_

pkMSP-1_42_ was purified by using nickel-NTA agarose resin which has a high binding affinity towards the N-terminal polyhistidine tag of the recombinant pkMSP-1_42_. ProBond^TM^ purification system (Invitrogen, USA) with hybrid condition was used. Cell pellet from a 50 ml pkMSP-1_42_ culture was dissolved and suspended in denaturing buffer (6 M guanidinium lysis buffer, pH 7.8) by using 16 ml guanidinium lysis buffer per gram of cell pellet. Cell lysate was sonicated on ice with three five-second pulses at high intensity for cell wall disruption. Purification column containing agarose resin was prepared under denaturing condition. The protein lysate was added to the column, washed twice with denaturing binding buffer (pH 7.8) containing 8 M urea and twice with denaturing wash buffer (pH 6.0) containing 8 M urea. Renaturation was carried out by washing four times using native wash buffer (pH 8.0) which contains 20 mM imidazole. The purified recombinant pkMSP-1_42_ was finally eluted with native elution buffer (pH 8.0) containing 250 mM imidazole. Concentration of purified pkMSP-1_42_ was determined by using Bradford Assay kit (Bio-Rad, USA).

### SDS-PAGE, Coomassie brilliant blue staining and Western blot

Non-purified and purified pkMSP-1_42_ were resolved by 12% SDS-PAGE under reducing and non-reducing conditions, and stained with Coomassie brilliant blue (Bio-Rad, USA). The separated proteins were also electrophorectically transferred onto polyvinylidene difluoride (PVDF) membranes (Bio-Rad, USA) and blocked overnight in tris buffered saline (TBS) containing 5% skimmed milk at 4°C. The membranes were probed with anti-Xpress^TM^ antibody (1:5000 dilution) with TBS containing 2.5% skimmed milk for one hour. The membranes were washed three times with TBS-T (TBS containing 0.2% Tween-20) and treated with biotin-labelled goat anti-mouse IgG (1:2,500 dilution) for one hour, followed by streptavidin-AP (1:2500 dilution) for one hour. Finally, the membranes were developed by chromogenic substrate NBT/BCIP. The colour was allowed to develop at room temperature in dark.

### Evaluation of purified pkMSP-1_42_ using serum samples in Western blot assay

The purified pkMSP-1_42_ was evaluated by Western blot assay using 189 sera of patients infected with *P. knowlesi* (n = 38), non-*P. knowlesi* malaria parasites (n = 29), non-malarial parasites (n = 47) and healthy donor (n = 75). PVDF membrane strips containing 60 ng of blotted pkMSP-1_42_ were incubated with the different serum samples, followed by biotin-labelled goat anti-human IgM + IgG + IgA (1:2500 dilution), streptavidin-AP, and finally NBT/BCIP.

### Evaluation of purified pkMSP-1_42_ by using serum samples in ELISA

The same 189 sera used in the Western blot assay were used in ELISA. Purified pkMSP-1_42_, 10 μg/ml, was coated on 96-well microtiter plates using 0.05 M sodium carbonate buffer, pH 9.6 at 4°C overnight. The wells were blocked with phosphate buffered saline (PBS) containing 1% bovine serum albumin for two hours at 37°C. The wells were washed three times with 0.1% PBS-T. Patient serum (1:80 dilution) was separately added into each well and incubated for one hour at 37°C. The wells were washed five times and peroxidase-labelled goat anti-human IgM + IgG + IgA (1:2,500 dilution) was added followed by one hour incubation at 37°C. The wells were washed five times with PBS-T and incubated with 3, 3′, 5, 5′-Tetramethyl Benzidine, TMB (Amresco, USA) for 30 minutes in dark. Stop solution 2 N H_2_SO_4_ was added to stop the reaction and absorbance at OD_450_ was measured. Samples were run in duplicates. The cut-off value was set at M_N_ + 2σ of the healthy donor serum group, where M_N_ is the mean absorbance (OD_450_) and σ is the standard deviation. Samples with absorbance values higher than M_N_ + 2σ were considered positive.

### Mice immunization

Six to eight-week old female BALB/c mice were used for immunization (pkMSP-1_42_-immunized group and negative control group, n = 5 per group). Purified pkMSP-1_42_, 30 μg, was mixed with adjuvant in a volume of 1:1 ratio and the mixture was injected into mice. Complete Freund’s Adjuvant (CFA) (Sigma, USA) was used in the prime boost and Incomplete Freund’s Adjuvant (IFA) (Sigma, USA) was used in the subsequent boosters. Booster was given on days 14 and 21 post-immunization. All injections were given subcutaneously. Serum of each mouse was collected at day 0, 7, 14, 21 and 31 post-immunization. Mice in the negative control group were injected with purified non-recombinant protein pRSET A.

### Measurement of cytokine levels in mice

Mice were sacrificed ten days after the second booster and their spleen cells were harvested and purified. The cells were grown in tissue culture grade, flat-bottom, 96-well microtitre plates (TPP, Switzerland), with total cells of 2 × 10^5^ per well. Stimulator purified pkMSP-1_42_ (30 μg/ml) was added. The plates were placed in 5% carbon dioxide (CO_2_) incubator at 37°C and the cells were allowed to grow for 65 hours. The plates were then centrifuged at 2,000 rpm for 20 minutes. Cell supernatants were collected and the levels of cytokine interleukin-2 (IL-2), interleukin-4 (IL-4), interleukin-10 (IL-10) and interferon-gamma (IFN-γ) in the supernatants were determined using ELISA kits (Thermo Scientific, USA) following the manufacturer’s instruction. Mann–Whitney statistical test was performed to determine whether increase of cytokine levels was significant.

### Antibody characterization and IgG subclass distribution

pkMSP-1_42_-immunized mice sera were analysed by Western blot assay for detection of antibody against pkMSP-1_42_. Purified pkMSP-1_42_, 350 ng, was blotted on PVDF membrane strips and incubated with mice sera collected at different time points. Level of IgM and IgG, IgG isotype distribution and endpoint titre of mice sera were determined by ELISA using purified pkMSP-1_42_ as coating antigen. Biotin-labelled anti-mouse IgM and IgG were used to determine the IgM and IgG level, respectively. Peroxidase-labelled anti-mouse IgG1, IgG2a, IgG2b and IgG3 were used for determination of IgG subclass distribution. For antibody endpoint titre determination, serial dilution was performed on the mice sera (1:400 – 1:819200 dilution) and detected by peroxidase-labelled anti-mouse IgM + IgG + IgA with TMB as substrate. Mice sera from the negative control group were used to determine the cut-off value as described before.

## Results

### Cloning, expression and purification of recombinant pkMSP-1_42_

**Figure 1:**
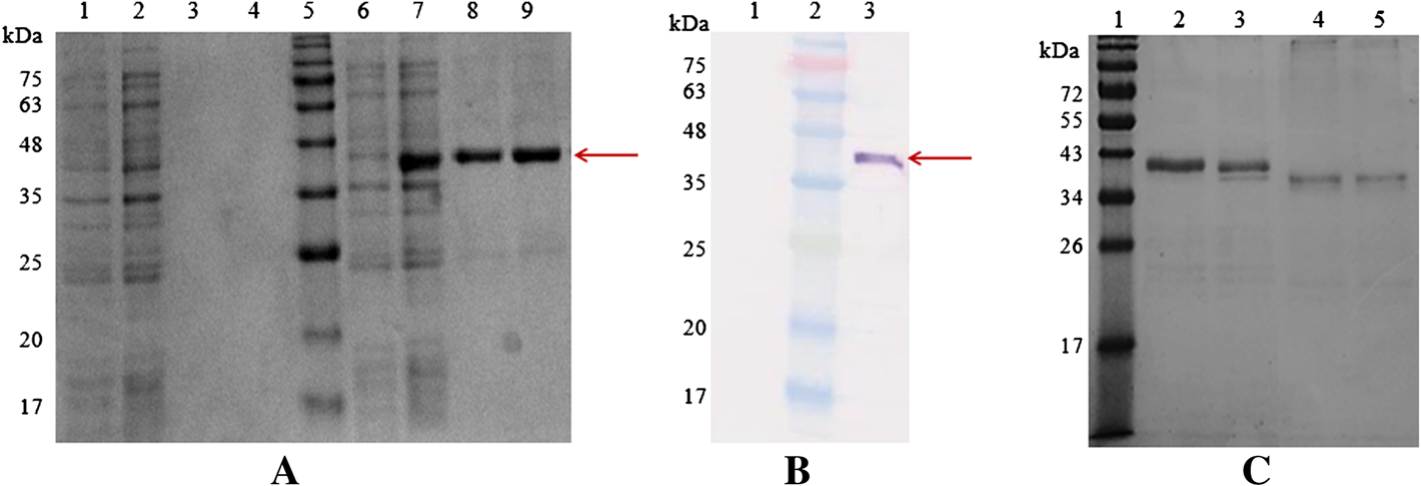
**Recombinant pkMSP-1**_**42 **_**produced in *Escherichia coli *expression system and purified with ProBond**^**TM **^**purification system.** The pkMSP-1_42_ with a size of ~42 kDa (arrows) was detected in **(A)** Coomassie brilliant blue stained SDS gel, and **(B)** Western blot probed with anti-Xpress^TM^ antibody. **(A)** Lanes 1 and 2, pRSET A at 0 and 2 hour respectively; lanes 3 and 4, purified pRSET A; lanes 6 and 7, recombinant pkMSP-1_42_ at 0 and 2 hour respectively; lane 8 and 9, purified pkMSP-1_42_. **(B)** Lane 1, purified pRSET A; lane 3, purified pkMSP-1_42_. **(C)** Lanes 2 and 3, purified pkMSP-1_42_ under reducing condition with sample boiling and without boiling respectively; lanes 4 and 5, purified pkMSP-1_42_ under non-reducing condition with sample boiling and without boiling respectively. Lane 5 in **(A)**, lane 2 in **(B)** and lane 1 in **(C)** were Prestained Broad Range Protein Marker.

The *P. knowlesi MSP-1*_*42*_ gene (969 bp) was amplified from genomic DNA by PCR. The amplified fragment was confirmed as *P. knowlesi MSP-1*_*42*_ gene through nucleotide sequencing and the deduced amino acid sequence. The recombinant plasmid pkMSP-1_42_ showed protein expression with molecular mass of ~42 kDa (Figure 1A, lanes 6 and 7). pkMSP-1_42_ was expressed as inclusion bodies which were solubilized under denaturing conditions by using denaturing guanidinium lysis buffer. Purified pkMSP-1_42_ showed a distinct band of ~42 kDa (Figure 1A, lanes 8 and 9 and Figure 1B, lane 3) which was absent in the purified plasmid control (Figure 1A, lanes 3 and 4 and Figure 1B, lane 1). The purified pkMSP-1_42_ was observed to have a molecular mass of ~42 kDa under reducing (Figure 1C, lanes 2 and 3) and ~39 kDa under non-reducing conditions (Figure 1C, lanes 4 and 5). The concentration of the purified pkMSP-1_42_ obtained was 1.0 mg/ml.

### Evaluation of purified pkMSP-1_42_ by using Western blot assay and ELISA

In the Western blot assay (Additional file 1), pkMSP-1_42_ reacted with 33/38 (86.8%) of knowlesi malaria serum samples and 28/29 (96.6%) of non-knowlesi malaria serum samples. Therefore, the overall sensitivity of pkMSP-1_42_ for malaria detection was 61/67 (91.0%). Three of the 122 non-*Plasmodium* parasitic infection and healthy donor sera reacted with pkMSP-1_42_, giving a specificity of 119/122 (97.5%). In ELISA, pkMSP-1_42_ reacted with 34/38 knowlesi malaria serum samples, thus giving sensitivity of 89.5% for knowlesi malaria detection. Twenty-seven of the 29 (93.1%) non-knowlesi malaria serum samples reacted with pkMSP-1_42_. Therefore, the overall sensitivity for detection of malarial infection was 61/67 (91.0%). The specificity of ELISA was 113/122 (92.6%). Evaluation of pkMSP-1_42_ with patient sera by using Western blot assay and ELISA is summarized in Table 1.

**Table 1:** **Evaluation of pkMSP-1**_**42 **_**with patient sera in Western blot assay and ELISA**

**Human sera group**	**Number of sera tested**	**Western blot**		**ELISA**	
		**Positive**	**Negative**	**Positive**	**Negative**
**A.** ***P. knowlesi***	38	33	5	34	4
**B. Non- *P. knowlesi *** **human malaria**					
i. *P. vivax*	15	14	1	14	1
ii. *P. falciparum*	13	13	0	12	1
iii. *P. ovale*	1	1	0	1	0
**C. Non-malarial parasitic infection**					
i. Filariasis	4	1	3	0	4
ii. Amoebiasis	16	2	14	4	2
iii. Cysticercosis	13	0	13	2	11
iv. Toxoplasmosis	11	0	11	1	10
v. Toxocarasis	3	0	3	1	2
**D. Healthy donor**	75	0	75	1	74

### Cytokine profiles in mouse

Cytokine secretion profiles of mice immunized with pkMSP-1_42_ were determined by ELISA. From the results, IFN-γ, IL-2, IL-4 and IL-10 levels of pkMSP-1_42_-immunized mice group were all significantly higher than those of the negative control group (*P* < 0.05) (Table 2).

**Table 2:** **Cytokine profiles of mice immunized with pkMSP-1**_**42**_

**Antigen**	**IL-2**	**IL-4**	**IL-10**	**IFN-γ**
pRSET A	75.8 (60.0-86.1)	3.4 (0.7-6.8)	89.9 (82.9-131.8)	165.7 (0.0-411.9)
pkMSP-1_42_	280.0 (268.1-287.0)* *P* = 0.008	91.0 (56.6-146.2)* *P* = 0.008	156.7 (136.8-250.8)* *P* = 0.016	2262.0 (1,515.0-2,437.0)* *P* = 0.008

Values shown are median (interquartile range). IL-2, interleukin-2; IL-4, interleukin-4; IL-10, interleukin-10; IFN-γ, interferon-gamma. Concentration of cytokines in pg/ml. *P < 0.05.

### Antibody characterization and IgG isotype distribution

**Figure 2:**
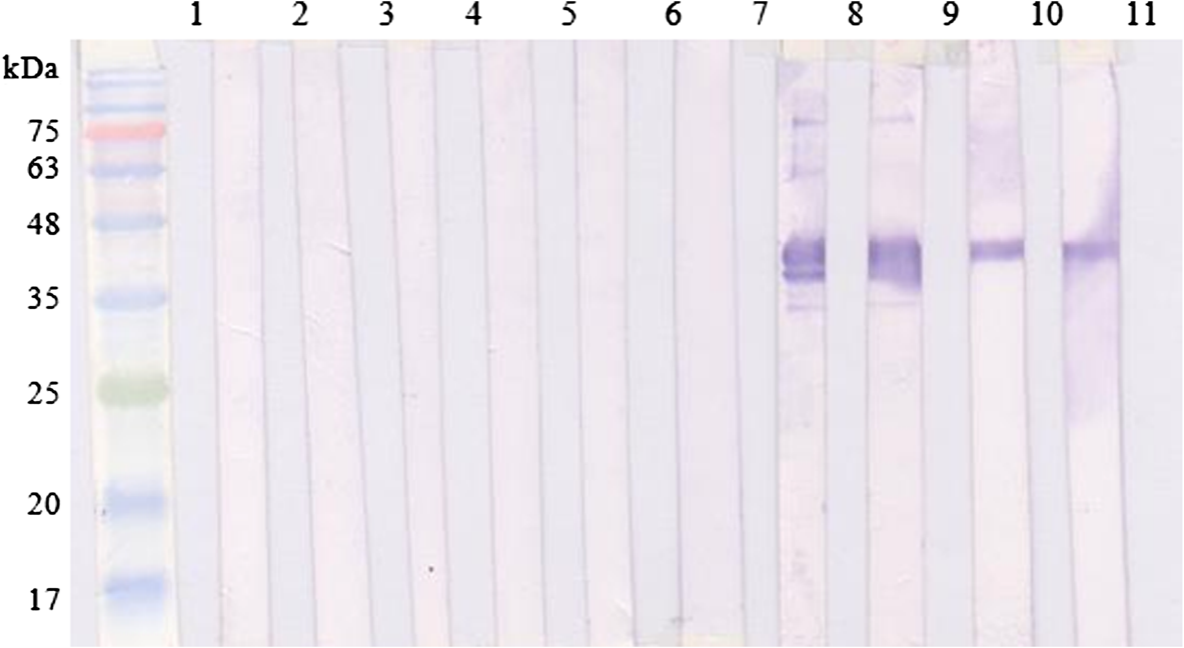
**Anti-pkMSP-1**_**42 **_**antibody detected in pkMSP-1**_**42**_**-immunized mice.** Lanes 2–6, sera of mice injected with non-recombinant protein pRSET A at day 0, 7, 14, 21 and 31 post-immunization; lanes 7–11, sera of mice injected with purified pkMSP-1_42_ at day 0, 7, 14, 21 and 31 post-immunization. Lane 1 contained protein size standards.

**Figure 3:**
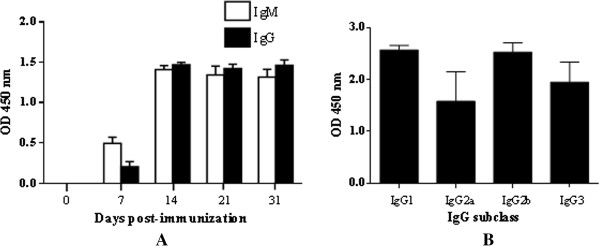
**Immune responses in pkMSP-1**_**42**_**-immunized mice. (A)** IgM and IgG levels in pkMSP-1_42_-immunized mice. Both IgM and IgG were detected at day 7 post-immunization and the levels increased throughout the whole immunization period. **(B)** IgG isotype-specific antibody levels. The IgG isotype distribution in pkMSP-1_42_-immunized mice was IgG1 > IgG2b > IgG3 > IgG2a.

Antibody responses in mice towards pkMSP-1_42_ at different time points were analysed. Western blot strips showed that antibody against pkMSP-1_42_ was detected one week after prime boost. pkMSP-1_42_ reacted with pkMSP-1_42_-immunized mice sera at day 7, 14, 21 and 31 post-immunization. No reactivity was observed in the negative control mice sera (Figure 2). ELISA results indicated that both IgM and IgG were detected in pkMSP-1_42_-immunized mice sera one week after prime boost. IgM level slightly decreased from day 14 until day 31 post-immunization. IgG level was relatively lower than IgM at first week after prime boost, yet high response was detected at day 14 post-immunization and continued to rise until day 31 post-immunization (Figure 3A). The predominant IgG isotype was IgG1, followed by IgG2b, IgG3, and IgG2a (Figure 3B). pkMSP-1_42_ induced high antibody response with the endpoint titre ranging between 1:204,800 and 1:819,200.

## Discussion

MSP-1_42_ of several *Plasmodium* sp. has been demonstrated to be immunogenic and able to elicit protective immunity [12,39]. MSP-1_42_ is non-glycosylated and this is crucial for its immunogenicity, as glycosylated form of milk-derived MSP-1_42_ secreted by transgenic mice does not confer protection against malaria during *Plasmodium* challenge [40]. The *E. coli* expression system was chosen in the present study due to its simplicity of techniques, cost-effectiveness and high efficiency of expression of non-glycosylated protein [31,41]. Hybrid condition was chosen for protein purification to preserve the protein structure and activity. The pkMSP-1_42_, which was expressed as inclusion bodies, was solubilized under denaturing buffer, then washed and eluted with native wash buffer and native elution buffer respectively to refold the protein. Purified pkMSP-1_42_ appeared to have a smaller molecular mass in non-reducing condition compared to reducing condition. The shift in mobility upon reduction in SDS-PAGE indicated the presence of disulfide linkages in pkMSP-1_42_ and hence pkMSP-1_42_ was likely refolded after purification.

In the present study, data showed high sensitivity (> 90%) of pkMSP-1_42_ to detect malarial infection in both Western blot and ELISA. Hence, this suggests that pkMSP-1_42_ is suitable as antigen in both assays for serodetection of malarial infection. The specificity of ELISA was relatively lower than that of the Western blot assay. This discrepancy might be due to the borderline activity of some patients’ sera in the ELISA. Suchankova *et al.* obtained positive results in ELISA but yet negative in Western blot when comparing the two assays for detection of human papillomavirus antibody. They suggested that the borderline activity of the sera, in which some of the serum sample OD absorbance values fall just above the cut-off value, led to the discrepancies in results [42]. Similarly, in the present study, some of the non-*Plasmodium* and healthy donor sera had OD absorbance values just slightly higher than the cut-off value, thus giving false positive results. Nonetheless, ELISA is useful as it can measure the titre of antibody compared to the qualitative detection of Western blot assay.

A few of the malaria infected patient sera did not react with pkMSP-1_42_ in Western blot and/or ELISA and this could be explained by the genetic diversity of MSP-1. *Plasmodium* MSP-1 exhibits extensive sequence diversity among isolates and host immune selective pressure could be one of the reasons that lead to the polymorphism [43,44]. *Plasmodium knowlesi* MSP-1 comprises of five conserved and four variable domains, which the conserved domains subjected to nucleotide substitutions and exhibited allelic dimorphism, while three of four variable domains contained complex repetitive sequence motifs which lead to extensive sequence and size variation. Besides, microheterogeneity comprising amino acid substitutions causing different alleles was observed in *P. knowlesi* MSP-1_33_ epitopes [45]. Presence of sequence diversity in these epitopes may alter the immunological recognition of the epitopes and hence benefit the parasite survival by evasion of host immune response. For instance, Bergmann-Leitner *et al.* demonstrated that the *P. falciparum* anti-MSP-1_19_ antibodies were allele-specific during inhibition of merozoite invasion and parasite growth [46]. Therefore, antibodies in some of the malaria infected patient sera in the present study could be unable to detect the variant epitopes on pkMSP-1_42_, which led to negative reactivity.

*Plasmodium knowlesi* MSP-1_33_ (pkMSP-1_33_) was evaluated with similar patient serum samples in the previous study [47]. The sensitivity of pkMSP-1_42_ for detection of malarial infection was similar with pkMSP-1_33_ in Western blot (both >90%), but higher compared to pkMSP-1_33_ in ELISA. pkMSP-1_42_ consisted of both MSP-1_33_ and MSP-1_19_ regionss. Previous studies showed that human sera from malaria-endemic areas demonstrated strong MSP-1_19_ reactivity [48] and MSP-1_19_ fragment consists of several immunodominant B cell epitopes which are important to induce protective anti-MSP-1_19_ antibodies [49]. Therefore, these epitopes could be recognized by specific anti-MSP-1_19_ antibodies in the sera of malaria-infected patients when pkMSP-1_42_ was used as antigen, but not pkMSP-1_33_.

pkMSP-1_42_ reacted with most of the non-knowlesi malaria sera and this could be due to serological cross-reactivity. Previous studies demonstrated that serum cross-reactivity could occur among malaria patients [50-55]. Furthermore, almost similar level of protection could be induced in immunized animals during challenge with heterologous *Plasmodium* parasite due to their high homology of antigens [56]. Sera from patients infected with *P. falciparum* and *Plasmodium malariae* cross-reacted with recombinant *Plasmodium vivax* MSP-1 which has 42% sequence similarity with *P. falciparum* MSP-1 [57]. *Plasmodium knowlesi* MSP-1_42_ shares high amino acid similarity with MSP-1_42_ of *P. vivax* (84%), *P. falciparum* (59%) and *Plasmodium ovale* (70%). Hence, *P. knowlesi* MSP-1_42_ may share certain common B-cell epitopes with human *Plasmodium* species, leading to cross-reactivity.

It has been reported that previous infection with *P. vivax* could be one of the reasons for reactivity of recombinant *P. vivax* AMA-1 with *P. falciparum*-infected patient sera [58]. In the present study, pkMSP-1_42_ reacted with some non-knowlesi human malaria and non-*Plasmodium* parasitic infection sera. This could probably due to the patients’ previous exposure to *P. knowlesi*. It is known that antibodies generated against knowlesi infection can persist up to five years or longer [59].

In the present study, high IFN-γ and IL-2 levels in pkMSP-1_42_-immunized mice group indicated that Th1-driven immune response has been stimulated. IFN-γ is a key molecule in human anti-malarial host defense. It activates macrophages to kill malarial blood stage parasites by reactive oxygen and nitrogen intermediates, and induces macrophages to secrete monokines such as IL-1, IL-6 and TNF [60,61]. IFN-γ, which regulates the pro-inflammatory and Th1 responses, was detected during primary *P. knowlesi* infection in rhesus macaques [62]. IL-2 functions as T cell growth factor and promotes the functional properties of natural killer cells, B cells and macrophages.

The high level of IL-4, IL-10 and predominant IgG1 production in the pkMSP-1_42_-immunized mice group showed that Th2 response has also been stimulated. IL-10 is an anti-inflammatory cytokine which is secreted by activated Th2 cells. It down-regulates the production of pro-inflammatory IFN-γ and limits the potentially harmful inflammatory responses during malarial blood stage parasites infection in mouse [63,64]. A study on *P. knowelsi*-inoculated olive baboons reported association between increased levels of IL-4, IL-10, IgM and IgG with increased protection against knowlesi-infection [65]. In natural human infection, Cox-Singh *et al.* reported increase in the IL-10 level in knowlesi malaria patients with considerable parasitaemia. Therefore, they postulated that this anti-inflammatory cytokine plays a role in modulating the expected immune surge during merozoite reinvasion [66].

Cytokines produced by each subset promote the polarization process, which Th1 cells-produced cytokines that will down-regulate Th2 response, and *vice versa*[67]. The concentrations of IFN-γ and IL-10 have been noted to increase in *P. vivax*-infected individuals during natural infection [68]. Therefore, stimulation of Th1 and Th2 subsets upon pkMSP-1_42_ immunization is important as homeostasis between Th1/Th2 cells could achieve a balance regulation between pro-inflammatory and anti-inflammatory actions in the immune response.

The high titre of anti-pkMSP-1_42_ antibodies suggests that pkMSP-1_42_ was highly immunogenic. Four isotypes of IgG were detected in the pkMSP-1_42_-immunized mice. These IgG isotypes help to activate effector responses in different manners. Murine IgG1 binds to mast cell, subtypes IgG2a and IgG2b play a role in complement binding and antibody opsonization, while IgG3 is responsible for carbohydrate epitope recognition [69]. IgG2a is the dominant IgG isotype for modulating murine malaria parasitaemia [70]. Both cell-mediated and humoral immunity were elicited by pkMSP-1_42,_ and these findings support *P. knowlesi* MSP-1_42_ as a potential blood stage vaccine candidate. Similar results were reported in studies involving mice immunized with recombinant *P. falciparum* and *P. vivax* MSP-1_42_[71,72].

## Conclusion

Results from the present study suggest that *E. coli*-expressed pkMSP-1_42_ can be useful in general seroprevalence and seroepidemiological screening. Moreover, pkMSP-1_42_ was highly immunogenic and both T cell and B cell responses were elicited in mice. Therefore, pkMSP-1_42_ can serve as a candidate for malaria vaccine design, although further evaluation needs be carried out to validate its potential and limitations.

## Competing interests

The authors declare that they have no competing interests.

## Authors’ contributions

FWC carried out the expression and immunogenicity assays, and drafted the manuscript. RM performed microscopic analysis of infected blood samples. MYF and YLL performed the data analysis. All authors read and approved the final manuscript.

## Supplementary Material

Additional file 1 **Purified pkMSP-1**_**42 **_**was detected by patient sera infected with knowlesi malaria and non-knowlesi malaria.** Western Blot strips containing 60 ng of the purified recombinant pkMSP-1_42_ were tested with selected sera from different categories. Lanes 2–5, sera from patients infected with malaria: *P. knowlesi* (lanes 2 and 3), *P. falciparum* (lane 4), *P. vivax* (lane 5). Lanes 6–10, sera of patients infected with non-malarial parasites: filariasis (lane 6), amoebiasis (lane 7), toxoplasmosis (lane 8), cysticercosis (lane 9), toxocarasis (lane 10). Lane 11, healthy donor serum which served as negative control. Lane 1 contained protein size standards. Click here for file
