# Supplementary figures and images for: Immunogenicity of bacterial-expressed recombinant Plasmodium knowlesi merozoite surface protein-142 (MSP-142)
Source: Malar J. 2013 Dec 19;12:454. doi: 10.1186/1475-2875-12-454 (PMC3878241; doi:10.1186/1475-2875-12-454)

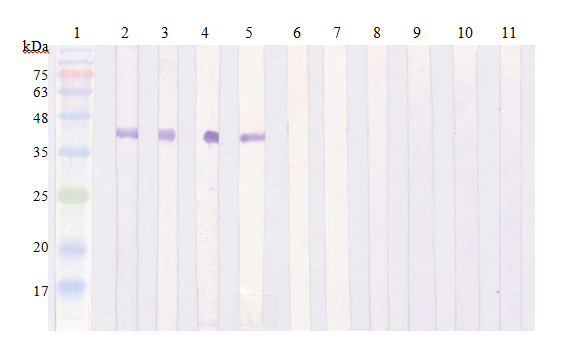

Supplement: Additional file 1 — Purified pkMSP-1 42 was detected by patient sera infected with knowlesi malaria and non-knowlesi malaria. Western Blot strips containing 60 ng of the purified recombinant pkMSP-142 were tested with selected sera from different categories. Lanes 2–5, sera from patients infected with malaria: P. knowlesi (lanes 2 and 3), P. falciparum (lane 4), P. vivax (lane 5). Lanes 6–10, sera of patients infected with non-malarial parasites: filariasis (lane 6), amoebiasis (lane 7), toxoplasmosis (lane 8), cysticercosis (lane 9), toxocarasis (lane 10). Lane 11, healthy donor serum which served as negative control. Lane 1 contained protein size standards. [file 1475-2875-12-454-S1.png]
